# An Examination of the Relationship between Lipid Levels and Associated Genetic Markers across Racial/Ethnic Populations in the Multi-Ethnic Study of Atherosclerosis

**DOI:** 10.1371/journal.pone.0126361

**Published:** 2015-05-07

**Authors:** Lucia Johnson, Jonathan Zhu, Erick R. Scott, Nathan E. Wineinger

**Affiliations:** 1 Scripps Translational Science Institute, La Jolla, CA, United States of America; 2 The Scripps Research Institute, La Jolla, CA, United States of America; Wake Forest University Health Sciences, UNITED STATES

## Abstract

Large genome-wide association studies have reported hundreds of genetic markers associated with lipid levels. However, the discovery and estimated effect of variants at these loci, derived from samples of exclusively European descent, may not generalize to the majority of the world populations. We examined the collective strength of association among these loci in a diverse set of U.S. populations from the Multi-Ethnic Study of Atherosclerosis. We constructed a genetic risk score for each lipid outcome based on previously identified lipid-associated genetic markers, and examined the relationship between the genetic risk scores and corresponding outcomes. We discover this relationship was often moderated by race/ethnicity. Our findings provide insight into the generalizability and predictive utility of large sample size meta-analyses results when leveraging data from a single population. We hope these findings will encourage researchers to investigate genetic susceptibility in more diverse populations and explore the source of such discrepancies. Until then, we caution clinicians, genetic counselors, and genetic testing consumers when interpreting genetic data on complex traits.

## Introduction

Lipids, including high-density lipoprotein (HDL) cholesterol, low-density lipoprotein (LDL) cholesterol, total cholesterol (TC), and triglycerides (TG), are frequently measured biomarkers used to evaluate an individual’s risk for cardiovascular disease[1,2]. Blood lipid concentrations are perhaps one of the most well characterized complex, polygenic traits as they are easily, precisely, and commonly measured, enabling massive meta-analyses of genome-wide association studies[3,4]; while unlike height[5], lipid levels can also be attributed to a major environmental component. Thus, the genetic architecture of lipids may provide better insight into the genetic etiology of other complex traits and common diseases related to human health. The largest meta-analysis of lipids have identified 157 loci at genome-wide significance levels which collectively account for approximately 12–14% of the phenotypic variance in lipid levels[4]. Importantly, while these studies each included over 100,000 participants of European ancestry, the number of non-Europeans was disproportionately low, which provided a limited opportunity to examine individual loci across diverse racial/ethnic populations. Carlson et al.[6] have shown that while the directions of effects at such loci are generally consistent across population, many display magnitude inconsistencies—suggesting researchers and clinicians need exercise care when applying genetic risk models to other racial/ethnic populations.

Here we expand upon a previous investigation of lipid levels[7] to investigate the predictive utility of previously identified lipid-associated genetic markers[3]. We used lipid and genetic data from 6,358 self-identified African Americans, Asian Americans, Caucasians, Hispanic American individuals from the Multi-Ethnic Study of Atherosclerosis[8] to calculate lipid genetic risk scores based on identified markers. Studies utilizing genetic risk scores have been recently popular as they have the potential to discriminate genetic susceptibility to complex traits from a moderate to large number of genetic markers[9–11]. We examined the relationship between estimated genetic risk and lipid levels across each population, and found that the strength of these relationships differed between populations. We discuss the discovery of this result in the context of massive research undertakings, caveats, and future directions for such inquiries.

## Results

**Fig 1:**
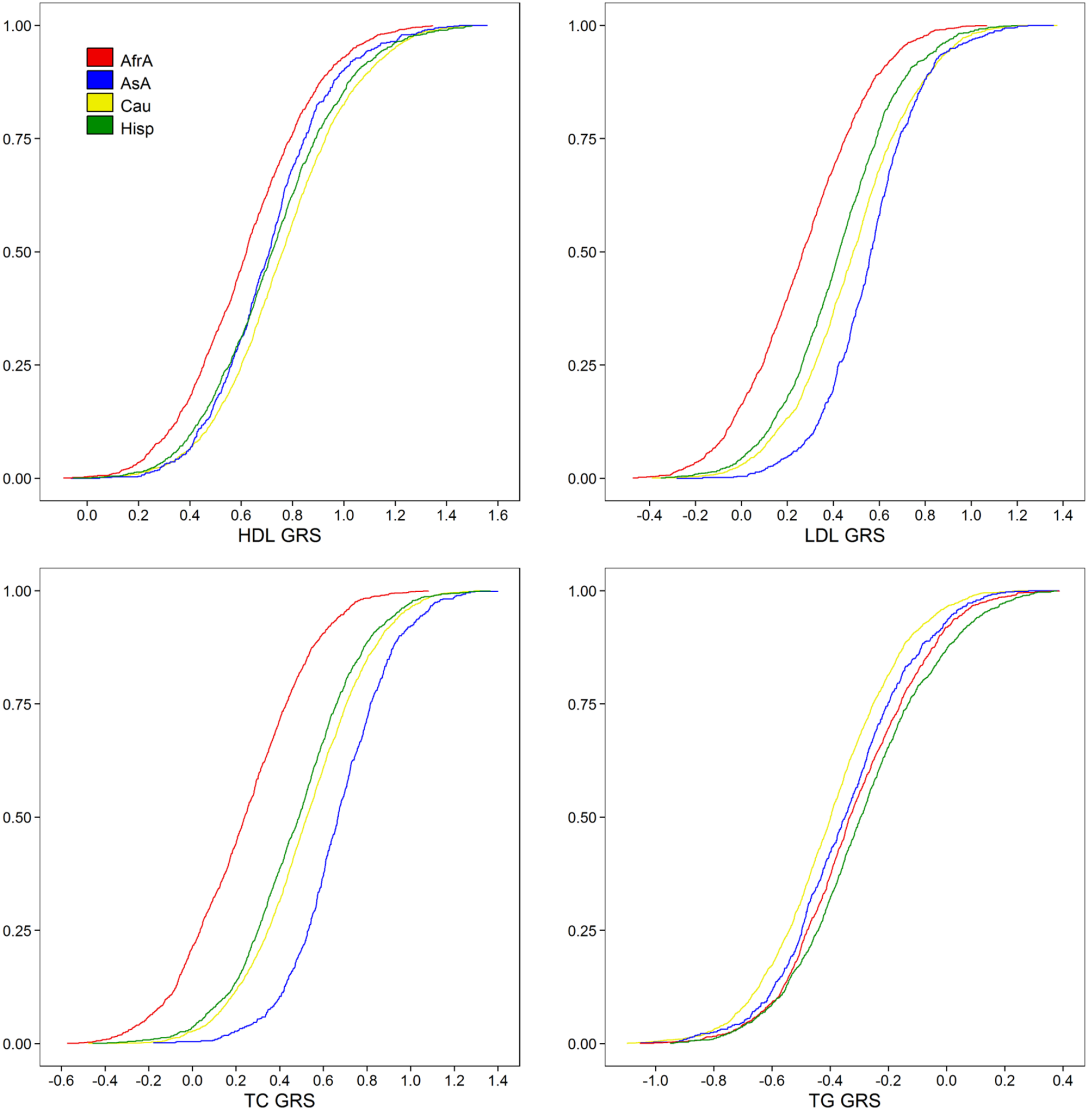
Cumulative density plots of observed genetic risk scores (GRS) for each trait. See Table 1 for reported means and standard deviation, and S1 Fig for unstacked histogram. Top left: HDL; top right: LDL; bottom left: total cholesterol; bottom right: log triglycerides. Red: African Americans; Blue: Asian Americans; Yellow: Caucasians; Green: Hispanics.

**Fig 2:**
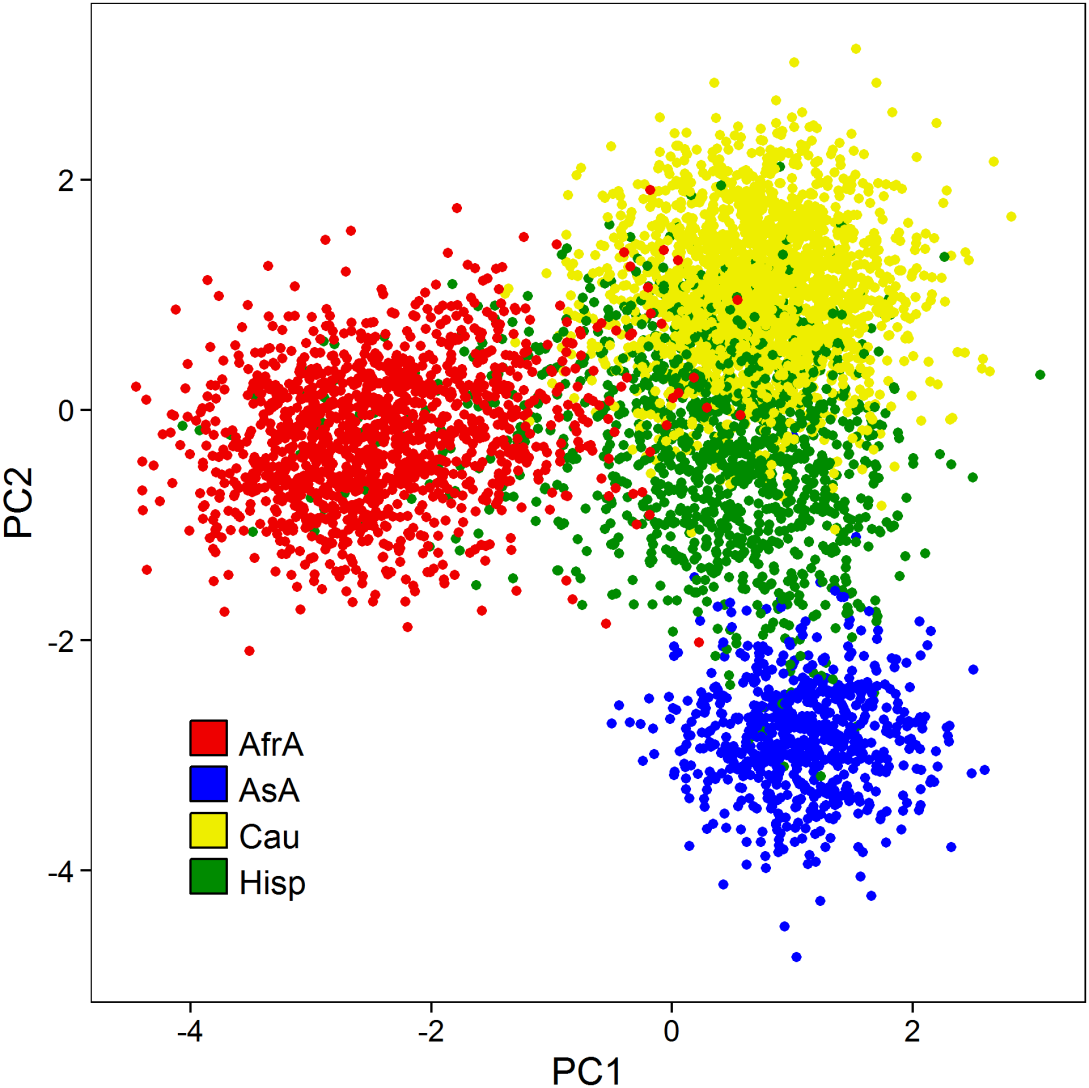
Principal component plot using all previously identified lipid-association genetic markers.

Self-reported race was associated with observed HDL (p<2.2 x 10^-16^), LDL (p = 2.82 x 10^-3^), TC (p = 5.34 x 10^-13^), and log TG (p<2.2 x 10^-16^). Genetic risk scores were calculated for all study individuals based on genome-wide significant markers previously identified in the Teslovich *et al*.[3] lipid meta-analysis. Self-reported race was also highly associated with each genetic risk score (all p<2.2 x 10^-16^). In general, African Americans had a substantially lower mean genetic risk score than other races with respect to HDL, LDL, and TC (Fig 1, S1 Fig), while observations across the other populations were less consistent. Caucasians had the highest mean HDL genetic risk, Asian Americans had the highest LDL and TC genetic risk, and Hispanics had the highest TG genetic risk. Caucasians had the lowest TG genetic risk (Table 1). Allele frequencies across all previously identified loci were highly differentiated across populations (largest p = 4.03 x 10^-4^, 96 of 101 markers p<10^-10^; S1 Table). Pairwise Pearson correlations between individuals at all previously identified markers are presented in Fig 2. Individuals of similar race/ethnicity appear to highly correlate with one another compared to across populations. Asian Americans and African Americans are noticeably distinct, suggesting reduced variation among loci in these populations. African Americans are most dissimilar to Caucasians, while Hispanics appear to share similarity to each other population. We observed similar results when we examined markers associated with each trait separately (S2 Fig).

**Table 1:** Mean (SD) genetic risk scores across self-reported racial groups.

	African Amer.	Asian Amer.	Caucasian	Hispanic
HDL	0.623 (0.246)	0.713 (0.225)	0.766 (0.249)	0.727 (0.255)
LDL	0.267 (0.263)	0.564 (0.213)	0.485 (0.256)	0.420 (0.249)
TC	0.233 (0.278)	0.673 (0.232)	0.523 (0.267)	0.481 (0.271)
TG	-0.316 (0.228)	-0.349 (0.222)	-0.398 (0.219)	-0.284 (0.239)

**Fig 3:**
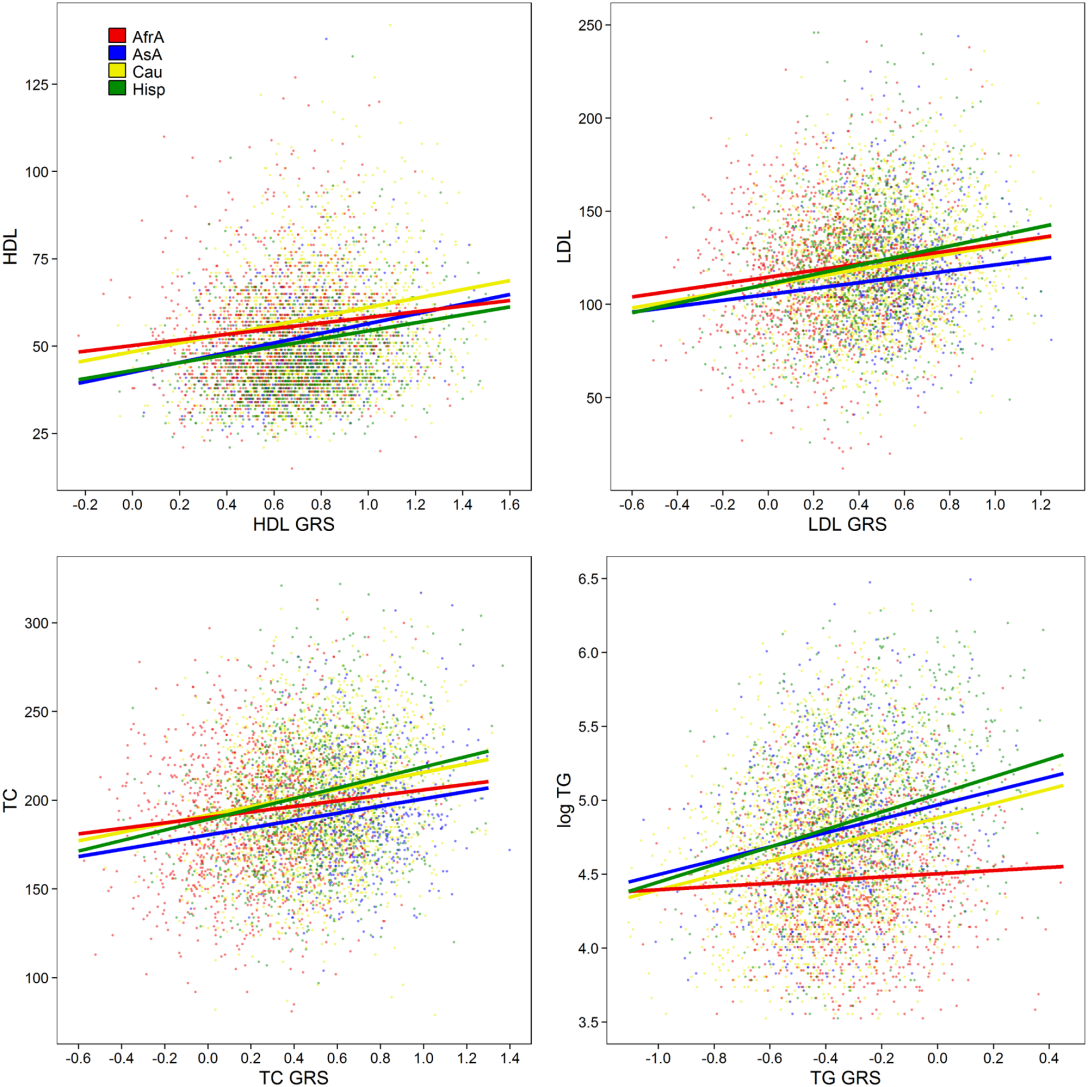
Linear association between genetic risk scores and the corresponding outcomes. Fitted lines correspond to the regression coefficients of a 50 year old female. Top left: HDL; top right: LDL; bottom left: total cholesterol; bottom right: log triglycerides. Red: African Americans; Blue: Asian Americans; Yellow: Caucasians; Green: Hispanics.

**Fig 4:**
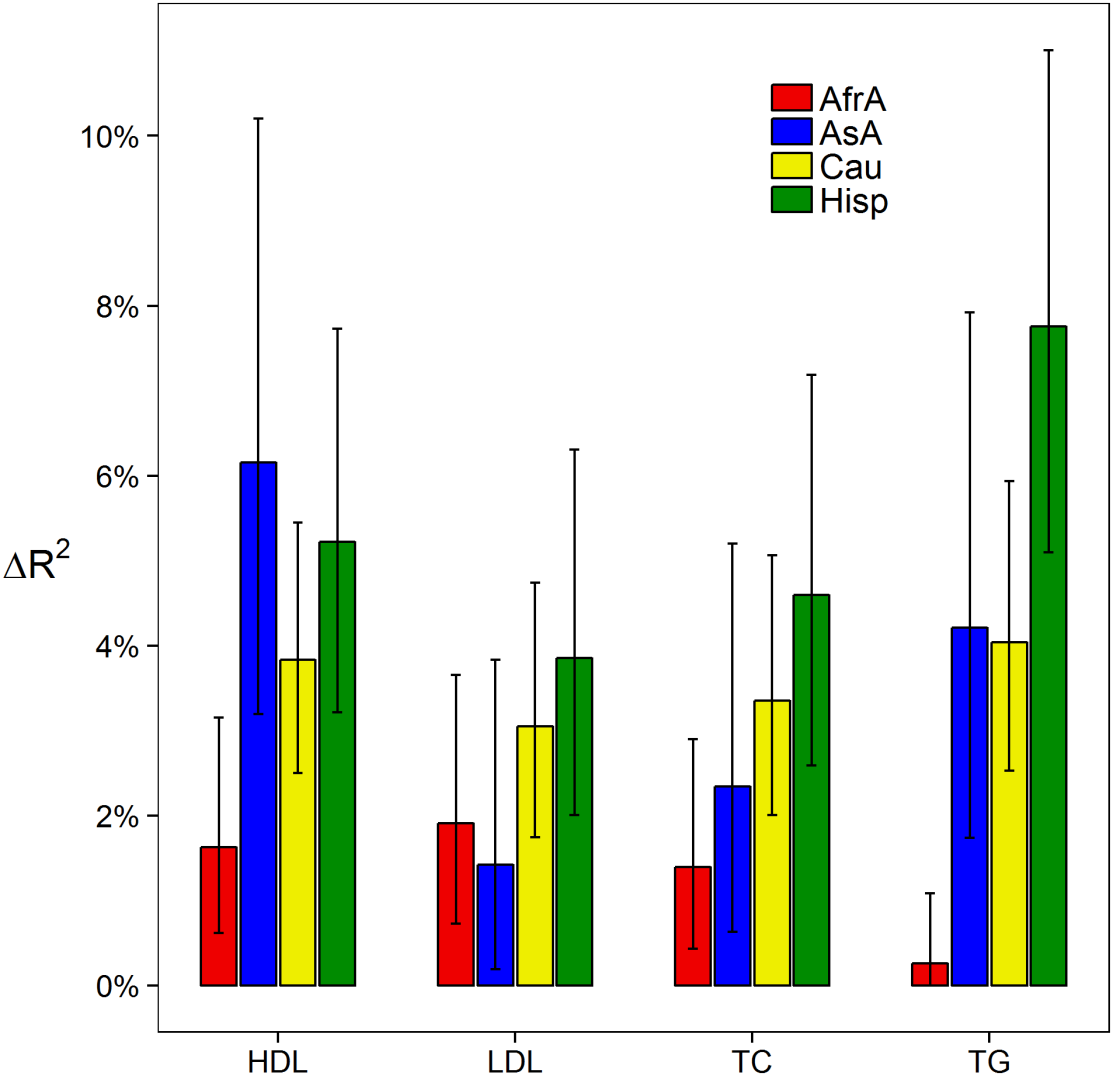
Added phenotypic variation explained by genetic risk scores over a covariate-only model. Error bars represent 95% confidence intervals. Red: African Americans; Blue: Asian Americans; Yellow: Caucasians; Green: Hispanics.

In nearly all instances we observed a significant linear correlation between genetic risk scores and the corresponding observed lipid level (p<0.001; Fig 3) with the exception of triglycerides in African Americans (p = 0.073). In most cases, the genetic risk scores resulted in a 3–6% increase in the *R* ^2^ estimate of the corresponding model in Caucasian and Hispanic populations. This estimate was noticeably lower in African Americans (0–2%; Fig 4). Indeed, we observed that race moderated the effect of the genetic risk score with HDL (p = 6.42 x 10^-3^), TC (p = 3.77 x 10^-2^), and TG (p = 1.48 x 10^-8^), though not LDL (p = 0.33).

We discovered in some cases that the non-corresponding genetic risk scores were jointly associated with alternative lipid outcomes after controlling for the corresponding genetic risk score (Table 2). For example, in Caucasians the LDL, TC, and TG genetic risk scores were jointly associated with HDL after controlling for HDL genetic risk (p = 4.36 x 10^-3^). African Americans had a general trend towards association across each outcome. This suggests other lipid-associated markers may be associated with the alternative trait in this population. However, only LDL and TC demonstrated significant associations across multiple racial/ethnic populations: three and two out of the four racial/ethnic populations, respectively. We also observed poor consistency of the individual effects. The HDL genetic risk score was positively correlated with TC in African Americans and Hispanics after controlling for other factors. In all other cases, no significant individual effects were observed across more than one population (S2 Table).

**Table 2:** Joint association results (p-values) of the non-corresponding genetic risk score with each lipid outcome controlling for the corresponding genetic risk score. For example, we can determine the contribution of non-HDL genetic risk scores to HDL above and beyond the HDL genetic risk score by testing the full model: HDL ~ HDL GRS + LDL GRS + TC GRS + TG GRS; against the reduced model: HDL ~ HDL GRS.

	African Amer.	Asian Amer.	Caucasian	Hispanic
HDL	0.08	0.62	4.36 x 10^-3^	0.62
LDL	5.48 x 10^-3^	0.29	1.96 x 10^-2^	1.22 x 10^-2^
TC	6.53 x 10^-3^	0.30	0.15	8.20 x 10^-3^
TG	0.05	0.23	0.57	0.17

**Fig 5:**
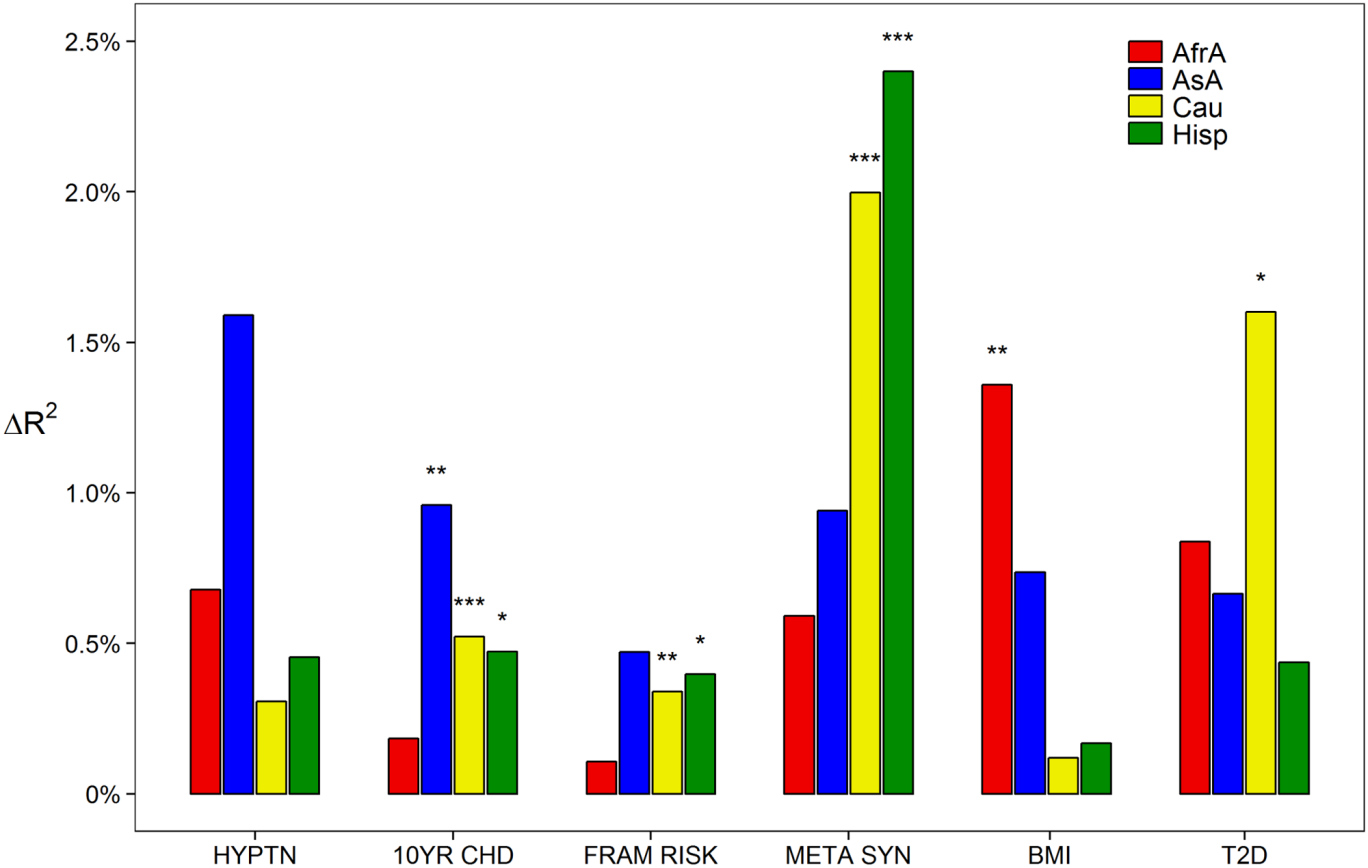
Added phenotypic variation explained by all genetic risk scores over a covariate-only model for non-lipid phenotypes. Asterisks represent p-values: * p<0.05; ** p<0.005; *** p<0.0005. Red: African Americans; Blue: Asian Americans; Yellow: Caucasians; Green: Hispanics.

Finally, we discovered that the lipid genetic risk scores were jointly associated with a number of clinical outcomes and risk factors in certain populations (Fig 5), though in all cases the predictive contribution was small (change in *R* ^2^ less than 2.5%). In all populations except African Americans, lipid genetic risk scores were associated with the National Cholesterol Education Program (NCEP) 10-year coronary heart disease risk. This appeared to be most strongly influenced by a negative association with the HDL genetic risk score (S2 Table). Genetic risk scores were also associated with the Framingham Risk Score (FRS) in Caucasians and Hispanics. We observed a negative association between FRS and HDL genetic risk in Caucasians, but not a clear distinction in Hispanics. Lipid genetic risk was most strongly associated with metabolic syndrome in Hispanics and Caucasians. Again, HDL genetic risk was negatively associated with the outcome; though TG genetic risk was positively associated as well. Interestingly, we only observed an association between lipid genetic risk and body mass index in African Americans. This appeared to be due to a positive association with LDL genetic risk and a negative association with TC genetic risk. Finally we observed an association with lipid genetic risk and Type 2 diabetes in Caucasians, though the contribution of each component was unclear. We did, however, observe a negative direction of association between Type 2 diabetes and TG genetic risk as others have observed[12,13] (one unit change in TG genetic risk score OR = 0.42, p = 0.17). We did not observe any significant association with hypertension.

## Discussion

We assessed the predictive accuracy of lipid genomic risk models generated from a large meta-analysis of individuals of European descent when applied to a racially diverse cohort from the Multi-Ethnic Study of Atherosclerosis. In nearly all cases, we found the genetic risk scores to be positively associated with the corresponding lipid measurement across racial/ethnic groups. While we observed excellent generalizability with respect to prediction of lipid levels and relevant health outcomes for self-reported Hispanics, we found the genetic risk models perform relatively poorly in African Americans. Thus, despite the fact that African Americans are disproportionately affected by hypertension[14], coronary heart disease[15], and myocardial infarction[16], our understanding of the genetic etiology and/or genetic risk factors of lipids, a major risk factor in cardiovascular and metabolic outcomes, remains relatively poor in this population.

Unfortunately, this suggests that the marker estimates obtained from genomic studies of over 100,000 individuals may translate poorly across all U.S. or world populations. This is in spite of the fact that the discovery of genetic risk variants in Caucasians and replication in non-Caucasians showed enrichment in the same direction of effect[3,6]. One cannot help wonder if the continuing tendencies towards larger and larger sample sizes from single populations (i.e. Caucasians) would be better served by examining a greater diversity of populations. Indeed, we observed substantial differences in allele frequencies at risk loci between populations—similar to those expected from 1000 Genomes[17]. While this notion has been embraced by funding agencies such as the National Institute of Health, we hope our findings encourage researchers to more fully characterize both the depth and breadth of any new discoveries.

There are a number of plausible explanations for our principal findings. Assuming the previously identified markers are truly causal, one possibility is that populations have unique genetic and allelic heterogeneity architecture. Thus, the contribution, direction, and effect of an identified locus may vary across populations. For example, markers or genes identified in Teslovich *et al*.[3] may not contribute to lipid levels in African Americans. If that is the case, then population-specific genetic risks and assessments should be explored as genetic background may modify the causal impact of the published loci. A second possibility is that the markers identified are non-causal, but in linkage disequilibrium (LD) with causal markers. Varying patterns of LD at these loci among populations will result in poor predictive utility across populations when only a single population is used in discovery and effect estimation. As demonstrated before[6], this is a reasonable explanation as African genomes have greater genetic diversity than other more recent populations due to immigration and genetic drift. One new approach to explore this claim would be to examine the genomes of African Americans, assign continent of origin (e.g. Africa or Europe) to chromosomal segments based on ancestral informative markers (AIMs), and test if the African segments are similar or dissimilar from European segments with respect the phenotype. Methods leveraging, instead of eliminating, population structure and admixture[18] should continue to be explored. Another possibility is the effect of the markers could be mediated by population-variable factors such as diet, lifestyle, or culture (i.e. gene-environment)[19] or by population-specific epistasis. In this instance, a more comprehensive exploration of the functional interaction between these factors should be undertaken.

Optimistically, the genetic risk scores explained a moderate amount of the trait variation (4–8%) in many instances. In the future we expect high dimensional models that incorporate wide clinical and genomic data will be more optimal when assessing common disease/complex trait risk/prediction. A critical, but common, assumption we made is that the effects of each marker are independent and each individual contribution can be summed together. This fails to account for non-independence between markers. Though we only modeled one marker per gene, thus eliminating the effects of LD, non-independence can also result from gene-gene interactions or population stratification. Concerning the latter, if common diseases and complex traits are a result of a polygenic architecture of thousands of causal variants[5], it is highly likely that a meaningful fraction of identified markers tag or are tagged by ancestry or population-specific markers (i.e. AIMs). In these instances, simply summing the effects of individual markers is potentially misleading. Approaches which estimate marker effects jointly, such as regularized regression procedures with cross-validation, or even multivariable regression in a discovery-estimation framework if sample sizes are sufficient are more ideal.

It is important that we reiterate the purpose of this study. The overarching goal of our work was to examine if the primary results from a large meta-analysis of over 100,000 individuals from a single population can generalize across all populations. Our work was not designed to reproduce the best possible genetic risk scores from results available from complementary sources (e.g. GWAS Catalog[20]) and/or contrast population-specific metrics. To do so would have created substantial bias in our comparisons across populations as it is well-recognized that the vast majority of genetic studies to date have been conducted primarily in individuals of European ancestry which tend to have larger sample sizes; and thus have a higher likelihood of replication, are less prone to publication bias, and have smaller variances on point estimates. Our approach, which used a standard set of markers, allowed us to make inference across populations concerning the combined contribution of these markers. Finally, we recognize that other markers in the genes previously identified may better in predict lipid levels across populations. We hope our results lead researchers with access to large amounts of data in different populations to instead highlight markers with more consistent, stable estimates[21] as opposed to markers with the smallest p-value in single populations.

For highly polygenic traits, our efforts to control, stratify, or eliminate population differences have, perhaps, limited our genomic understanding to an insufficient number of discoveries in only specific populations. Instead, we should explore the notion that many causal markers may have considerable allele frequency differences or variable effects across populations. If this is true, we will continue to fail to identify such variants by restricting our studies to uniform populations. Instead, genetically diverse admixed populations may reveal a wealth of genomic insight which may complement our understanding of why complex traits demonstrate such profound differences across populations.

### Limitations

We acquired genetic information on most of the markers used in this analysis through imputation. While the majority of markers were quite common (S1 Table), and thus likely display high accuracy (*R* ^2^>0.8) even in non-European populations[22], there is potential that imputation quality may be differ at these markers across populations. Unfortunately, as only a small number of the markers used to construct the genetic risk scores were genotyped in the MESA cohort (n = 16), we were unable to examine if the trends we observed were consistent across genotyped and imputed marker sets. Also, we chose to model race/ethnicity based on self-report as opposed to genetically derived ancestry. This was done to simplify results as opposed to being forced into modeling and discussions of effects based on percentages of worldwide populations, particularly in the admixed populations. It should be noted that using self-reported race/ethnicity is more conservative. Thus, we would expect similar analyses based on genetically derived ancestry to, at a minimum, show the same differences we observed.

## Methods

### Study participants

Genotype and phenotype data was obtained on study participants in the Multi-Ethnic Study of Atherosclerosis (MESA) Classic cohort (n = 6,358) through access via the database of Genotypes and Phenotypes (dbGaP; phs000209.v2.p1). MESA participants were genotyped on the Affymetrix Genome-Wide Human SNP Array 6.0. The MESA cohort and study design has been extensively characterized[8,23,24]. Individuals on any lipid-lowering medication (n = 1,018) were omitted from all analyses as was previously reported[3,4]. The remaining present study population included self-identified Caucasians (n = 2,063; 38.6%), African Americans (n = 1,355; 25.4%), Hispanics (n = 1,256; 23.5%), and Asian Americans of Chinese descent (n = 666; 12.5%). Demographic and relevant clinical characteristics are presented in S3 Table. Lipid levels are represented in mg/dL. This study was approved by the Scripps Health Institutional Review Board (IRB).

### Imputation

Prior to imputation, genetic markers were excluded which demonstrated high missingness (>0.05), failed Hardy—Weinberg equilibrium (*p*<0.0005), or had exceedingly rare alternative alleles (minor allele frequency <0.005). The remaining genetic data were pre-phased[22], and genome-wide imputation was performed on the resulting haplotypes using the default parameters in IMPUTE v2.2.2[25]. The 1000 Genomes Phase 1 integrated variant set haplotypes were used as the reference panel[26]. Genomes were divided into approximately 5 Mb segments (avoiding chromosome and centromere boundaries) with phasing and imputation calculated on each. The minimum information score among markers used to construct the genetic risk scores (see below) was 0.474; and thus no such markers were omitted based on this criteria.

### Genetic risk scores

Genetic risk scores were calculated for all study individuals based on markers previously identified in the Teslovich *et al*.[3] lipid meta-analysis (Fig 1, Table 1, S1 Fig). Genetic risk scores were constructed using a weighted allele-counting approach similar to that performed in a recent study[12]. For each previously identified marker and study individual, the observed allelic count, or allelic dosage in the case of imputed markers or missing genotypes, was multiplied by the corresponding reported effect (S4 Table). An individual’s genetic risk score was then calculated as the summation of these products across all such marker. This calculation was performed for each lipid trait: HDL (n = 46 markers), LDL (n = 37), TC (n = 52), and TG (n = 32). The previously reported marker rs1084651was not observed in the MESA cohort. Notably, only one marker (lead SNP) per gene was used to construct each score.

### Data analyses

Allele frequencies of all previously reported markers were calculated within self-reported racial/ethnic populations and allelic count/dosage was contrasted between populations via F-test (S2 Table). Principal component analyses across all races among previously reported markers were conducted (Fig 2). Pairwise Pearson correlation between individuals was conducted in Python version 2.7.1 and accompanying packages using genotypes at all previously identified markers and lipid-specific associated genetic markers (S2 Fig). In all other statistical analyses, age, age-squared, and sex were modeled as covariates as was similarly performed[3,4]. By design, continuous axes of ancestry (e.g. principal components) were omitted from all statistical modeling. Principal components are collinear with race/ethnicity, and therefore would have distorted any inference concerning race. Additionally, Teslovich et al.[3] reported very few markers displaying dissimilar effects between studies which modeled principal components and those that did not. Observed HDL, LDL, total cholesterol, and the logarithm of triglycerides were regressed on the corresponding genetic risk score, stratified by self-reported race (Fig 3). Increase in the variation explained by this model over a model that only included covariates was measured by the change in *R* ^2^ (Δ*R* ^2^; Fig 4). Self-reported race as a moderating factor on the effect of each genetic risk score (i.e. race x genetic risk score interaction) was tested. Observed lipid outcomes were also regressed on a multivariable model, which included all four genetic risk scores, and joint tests of association with the three non-corresponding genetic risk scores were performed (Table 2). Similarly, hypertension status, ten year coronary heart disease risk (NCEP), Framingham global cardiovascular disease risk, metabolic syndrome status, body mass index, and type 2 diabetes status were tested for association with all four genetic risk scores (jointly) using linear or logistic regression as appropriate. Results are presented as change in *R* ^2^ (discrete outcomes as change in Nagelkerke *R* ^2^) over a model that only included covariates (Fig 5). R version 3.1.1 was used for all analyses. All p-values are presented without multiple testing correction.

## Supporting Information

S1 Fig Unstacked histograms of observed genetic risk scores (GRS) for each trait. Top left: HDL; top right: LDL; bottom left: total cholesterol; bottom right: log triglycerides. Red: African Americans; Blue: Asian Americans; Yellow: Caucasians; Green: Hispanics.
(PNG)

S2 Fig Pairwise Pearson correlation between individuals at associated genetic markers: all markers (page 1), HDL (page 2), LDL (page 3), TC (page 4), TG (page 5). (DOCX)

S1 Table Allele frequencies of lipid-associated alleles in each population with respect to the A2 allele. p-values test equal allele frequencies across populations.
(CSV)

S2 Table Joint tests of association between all lipid genetic risk scores with lipid levels and clinical outcomes. The overall p-value assesses joint association and delta *R* ^2^ is the change in variation explained over a model only including age, age^2^, and sex covariates. Individual parameter estimates and corresponding p-values are presented.
(CSV)

S3 Table Demographic data of the study cohort by self-reported race and gender. HLD = high-density lipoprotein cholesterol; LDL = low-density lipoprotein cholesterol; TC = total cholesterol; log TG = logarithm (base *e*) triglycerides; HYPTN = hypertension (yes); 10YR CHD = National Cholesterol Education Program (NCEP) 10 year coronary heart disease risk; FRAM RISK = Framingham Risk Score (FRS); META SYN = metabolic syndrome (yes); BMI = body mass index; T2D = type-2 diabetes (yes).
(DOCX)

S4 Table Weights used to calculate genetic risk scores based on the allelic count/allelic dosage of A2 alleles. (CSV)
