# Supplementary figures and images for: An Examination of the Relationship between Lipid Levels and Associated Genetic Markers across Racial/Ethnic Populations in the Multi-Ethnic Study of Atherosclerosis
Source: PLoS One. 2015 May 7;10(5):e0126361. doi: 10.1371/journal.pone.0126361 (PMC4423846; doi:10.1371/journal.pone.0126361)

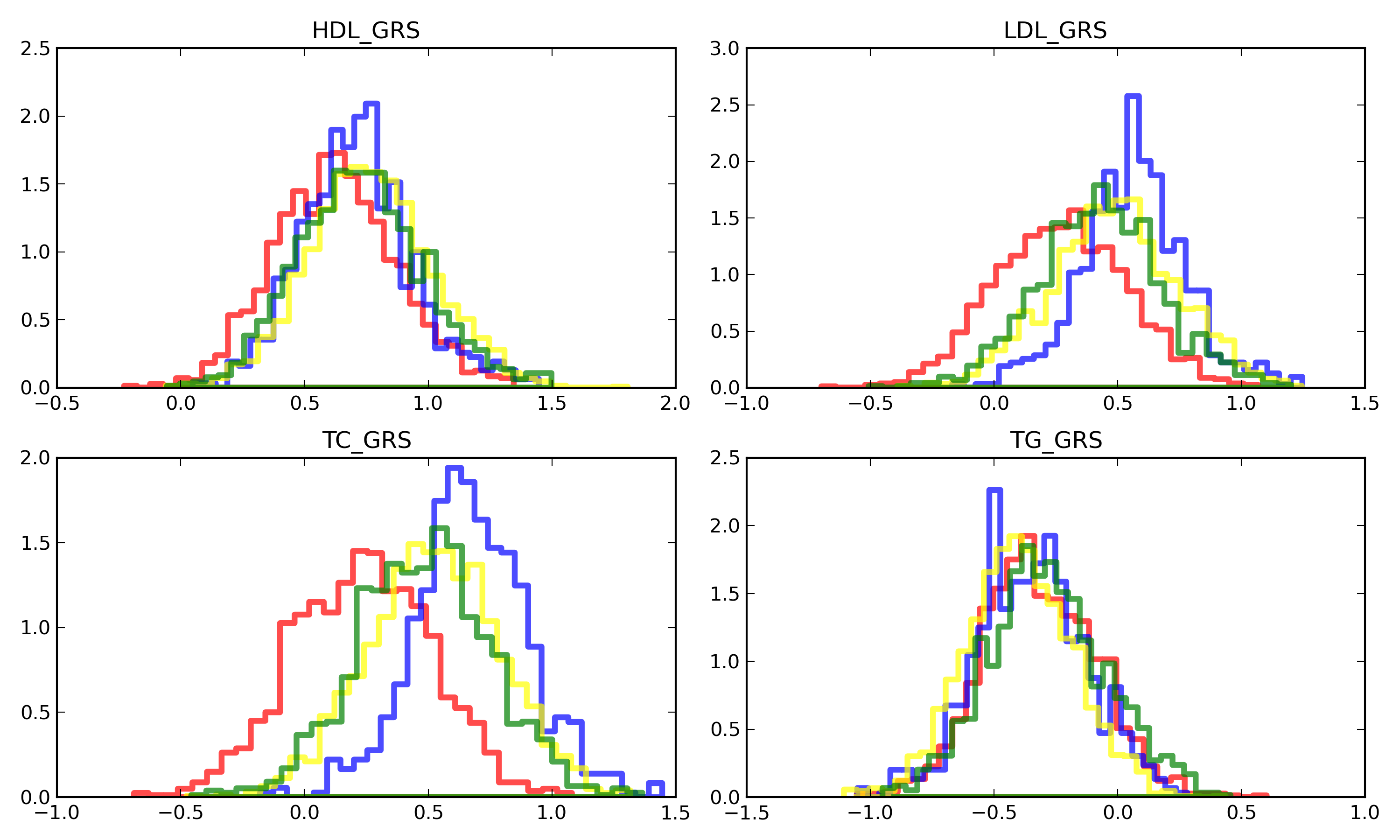

Supplement: S1 Fig — Top left: HDL; top right: LDL; bottom left: total cholesterol; bottom right: log triglycerides. Red: African Americans; Blue: Asian Americans; Yellow: Caucasians; Green: Hispanics. (PNG) [file pone.0126361.s001.png]

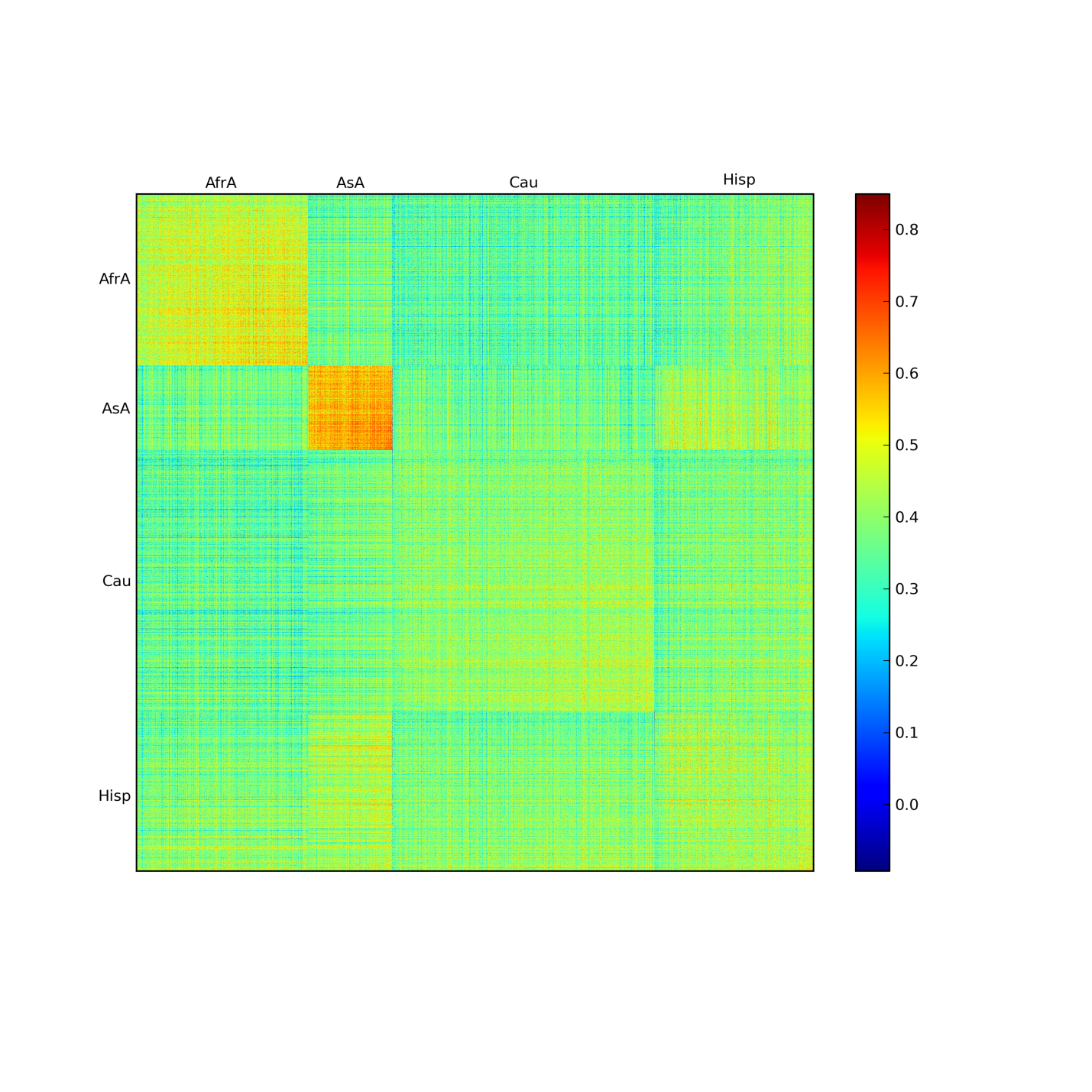

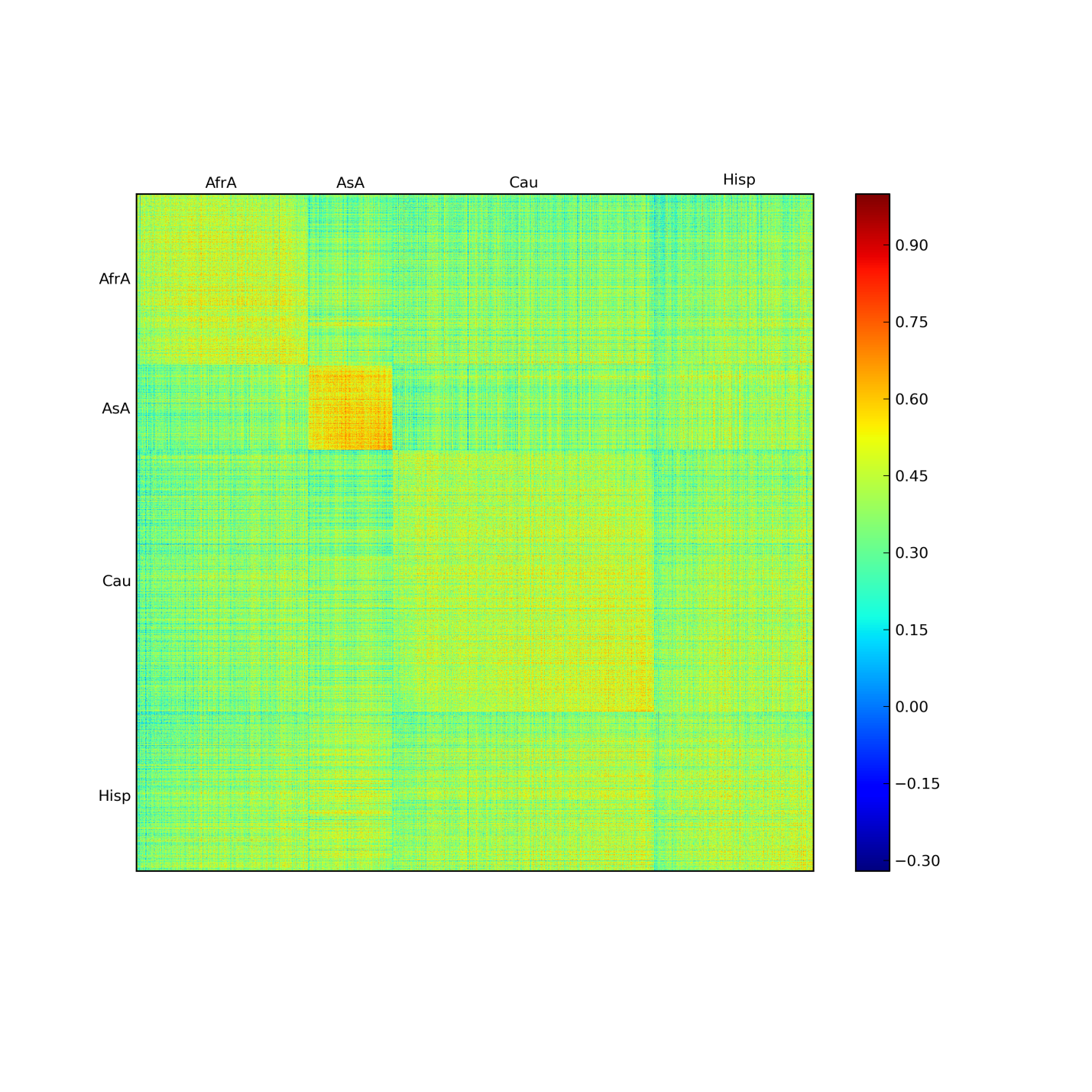


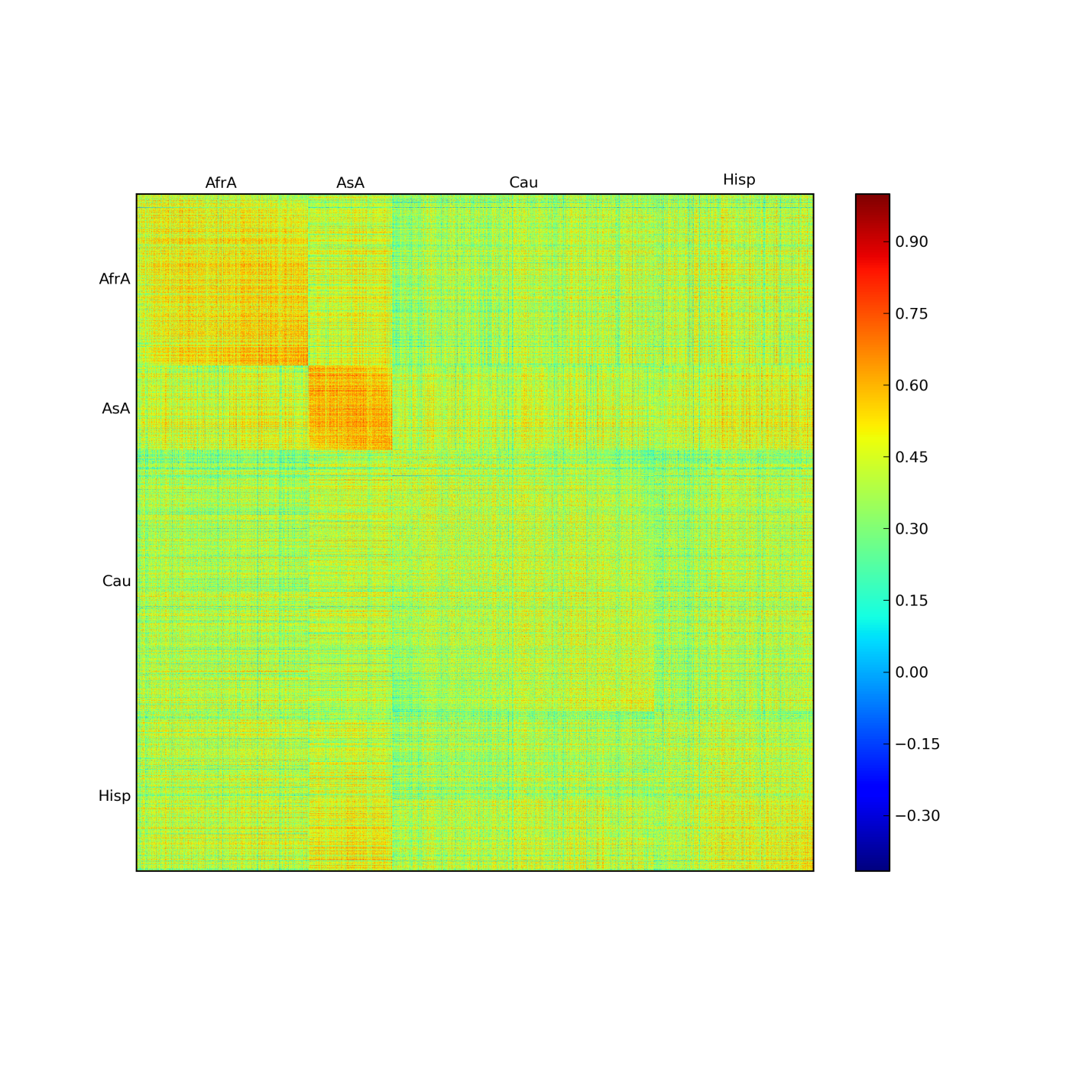


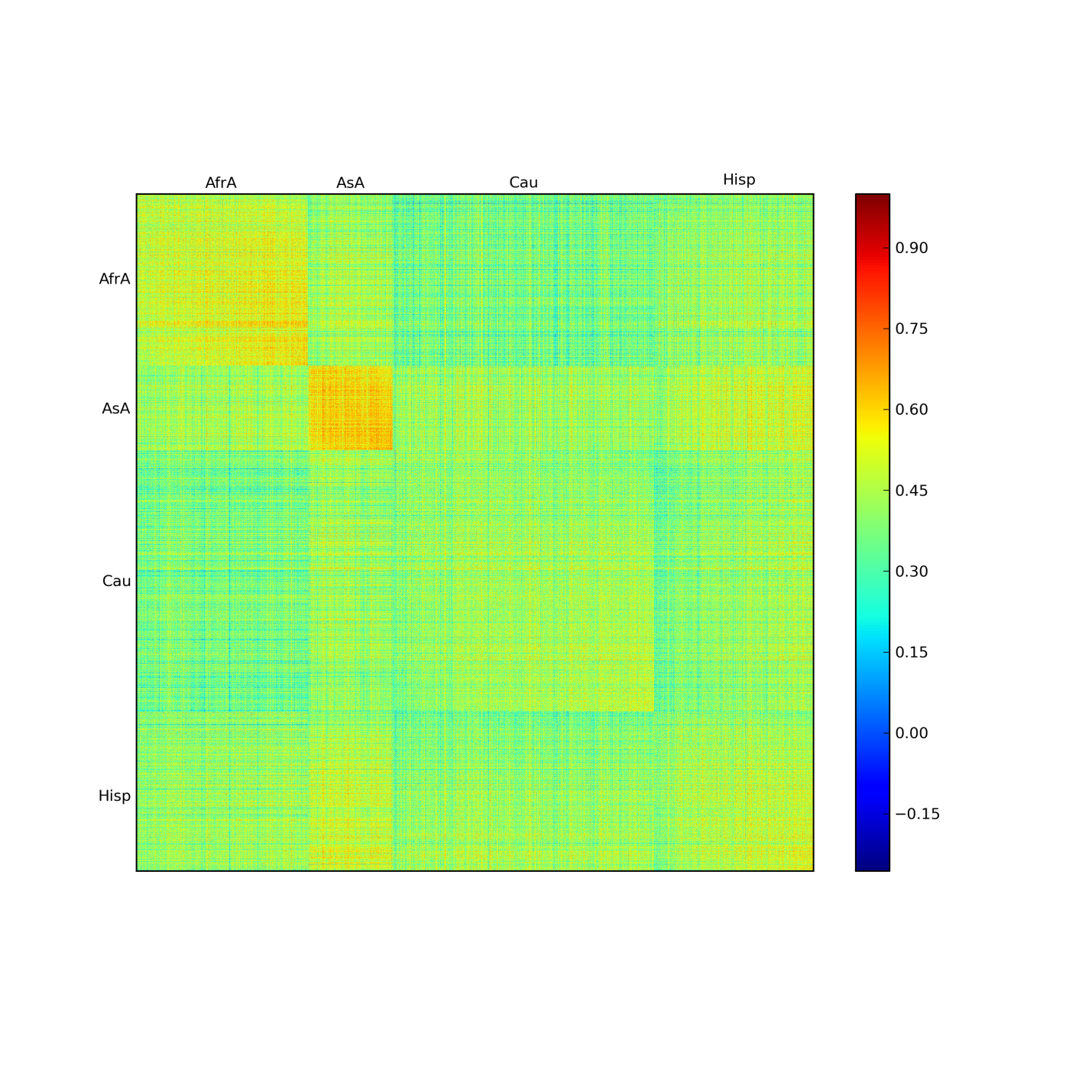


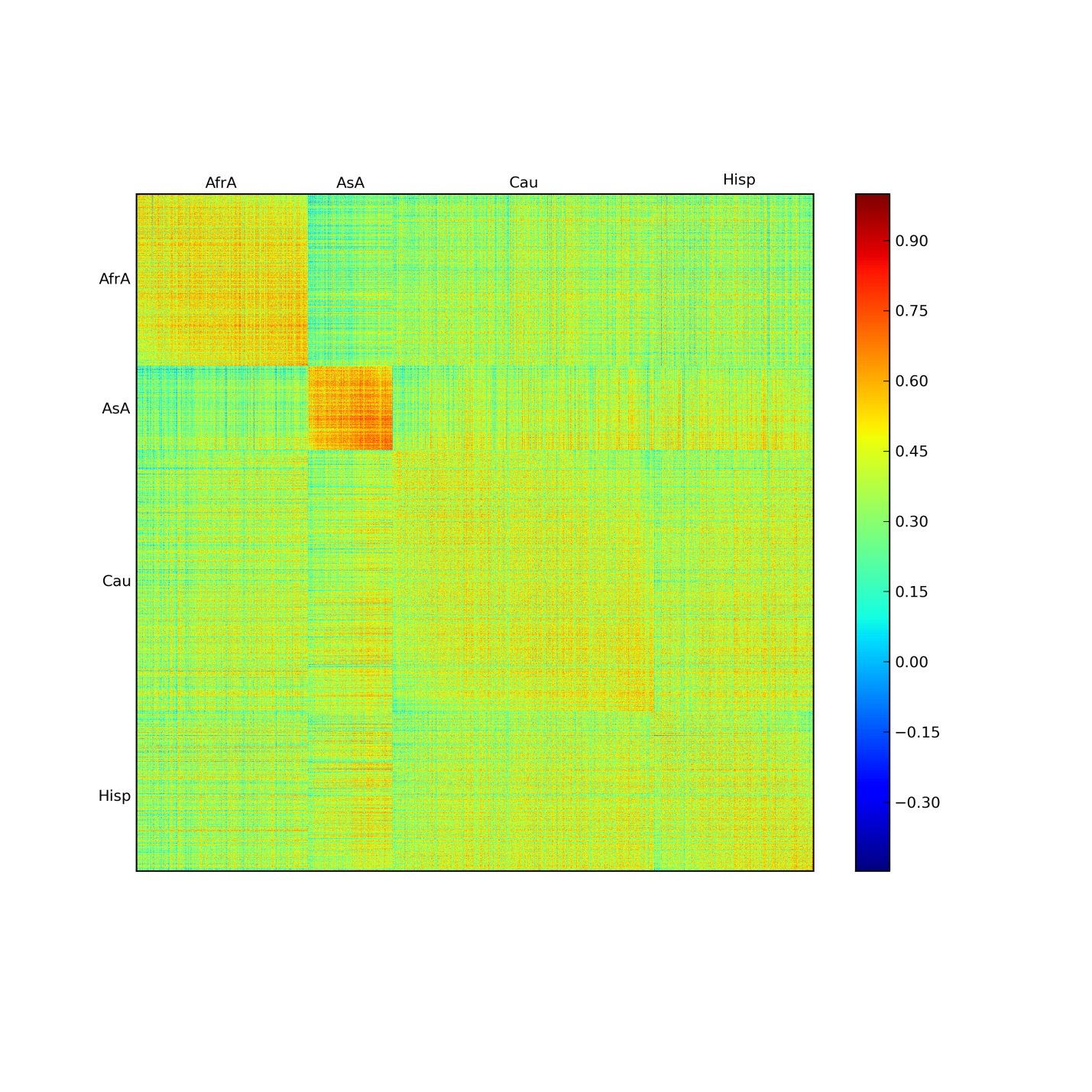

Supplement: S2 Fig — (DOCX) [file pone.0126361.s002.docx]
