# Supplementary material for: An Examination of the Relationship between Lipid Levels and Associated Genetic Markers across Racial/Ethnic Populations in the Multi-Ethnic Study of Atherosclerosis
Source: PLoS One. 2015 May 7;10(5):e0126361. doi: 10.1371/journal.pone.0126361 (PMC4423846; doi:10.1371/journal.pone.0126361)
Supplement: S3 Table — HLD = high-density lipoprotein cholesterol; LDL = low-density lipoprotein cholesterol; TC = total cholesterol; log TG = logarithm (base e) triglycerides; HYPTN = hypertension (yes); 10YR CHD = National Cholesterol Education Program (NCEP) 10 year coronary heart disease risk; FRAM RISK = Framingham Risk Score (FRS); META SYN = metabolic syndrome (yes); BMI = body mass index; T2D = type-2 diabetes (yes). (DOCX) [file pone.0126361.s005.docx]

|  | African Amer. (n=1,355) | | Asian Amer. (n=666) | | Caucasian (n=2,063) | | Hispanic (n=1,256) | |
| --- | --- | --- | --- | --- | --- | --- | --- | --- |
| Gender F/M | 729 (54%) | 626 (46%) | 332 (50%) | 334 (50%) | 1,098 (53%) | 965 (47%) | 642 (51%) | 614 (49%) |
| Age | 61.5 (10.1) | 61.9 (10.3) | 61.2 (10.5) | 61.8 (10.2) | 61.9 (10.4) | 62.3 (10.4) | 60.7 (10.4) | 60.6 (10.3) |
| HDL | 57.3 (15.9) | 46.9 (12.9) | 53.0 (13.1) | 45.5 (10.9) | 59.9 (16.1) | 45.2 (12.4) | 52.0 (13.3) | 42.2 (9.8) |
| LDL | 119.8 (33.6) | 116.2 (33.4) | 116.2 (28.4) | 118.6 (28.0) | 120.4 (30.8) | 120.6 (30.0) | 122.4 (32.7) | 121.2 (32.3) |
| TC | 197.0 (36.7) | 184.6 (35.2) | 197.2 (30.5) | 191.1 (30.8) | 205.5 (34.1) | 192.3 (34.9) | 204.1 (38.2) | 195.2 (36.2) |
| log TG | 4.48 (0.46) | 4.55 (0.50) | 4.82 (0.49) | 4.79 (0.52) | 4.69 (0.52) | 4.74 (0.53) | 4.87 (0.49) | 4.96 (0.53) |
| HYPTN | 431 (59%) | 330 (53%) | 119 (36%) | 111 (33%) | 384 (35%) | 339 (35%) | 269 (42%) | 216 (35%) |
| 10YR CHD | 0.045 (0.052) | 0.126 (0.071) | 0.037 (0.048) | 0.117 (0.065) | 0.038 (0.046) | 0.123 (0.065) | 0.044 (0.054) | 0.126 (0.067) |
| FRAM RISK | 11.6 (8.4) | 19.7 (9.3) | 9.3 (7.8) | 17.6 (9.3) | 8.8 (7.1) | 17.9 (9.1) | 10.9 (8.7) | 18.7 (9.3) |
| META SYN | 277 (38%) | 198 (32%) | 101 (30%) | 73 (22%) | 318 (29%) | 284 (30%) | 313 (49%) | 333 (47%) |
| BMI | 31.4 (6.5) | 28.5 (4.6) | 23.7 (3.4) | 23.9 (3.2) | 27.3 (5.9) | 27.9 (4.2) | 30.1 (5.7) | 28.7 (4.3) |
| T2D | 73 (10%) | 79 (13%) | 21 (6%) | 28 (8%) | 31 (3%) | 39 (4%) | 72 (11%) | 81 (13%) |
